# Psychosocial correlates with depressive symptoms six years after a first episode of psychosis as compared with findings from a general population sample

**DOI:** 10.1186/1471-244X-4-29

**Published:** 2004-10-05

**Authors:** Yvonne Forsell, Sonja Levander, Johan Cullberg

**Affiliations:** 1 Dep. of Public Health, Division of Social Medicine, Karolinska Institutet, S-171 76 Stockholm, Sweden; 2 Stockholm Center of Public Health, Unit of Mental health, Stockholm, Sweden

## Abstract

**Background:**

Depression is frequently occurring during and after psychosis. The aim of this study was to analyze if the psychosocial characteristics associated with depression/depressive symptoms in the late phase of a first episode psychosis (FEP) population were different compared to persons from the general population.

**Methods:**

A questionnaire was sent out to all individuals six years after their FEP and to a general population sample. Depressive symptoms were recorded using a self-rating scale, the Major Depression Inventory.

**Results:**

Formerly FEP persons had a higher representation of depressive symptoms/depression, unemployment, financial problems and insufficient social network. Depressive symptoms/depression were found to be associated with psychosocial problems. An age and gender effect was found in the general population, but not in the FEP sample. When the psychosocial characteristics were taken into account there were no association between having had FEP and depressive symptoms.

**Conclusions:**

The association between having been a FEP patient and depressive symptoms/depression disappeared when negative social aspects were taken into account.

## Background

Major Depression is the one of the most common psychiatric disorders and is frequently occurring in persons with psychotic disorders. Up to 25% of individuals with psychosis have this condition at some point during the course of their illness [1].

Insufficient social network, unemployment, living alone, financial problems and low social class are among the reported risk factors for depression in prospective studies [2-5]. Studies of characteristics associated with depression in psychosis have reported various results. Associations have been reported with negative as well as positive symptoms, medications and neuroleptic induced movement disorders [6-9]. In a study by Baynes et al, depressive symptoms were explored in a population of 120 patients with stable, chronic schizophrenia living in the community [10]. Patients who perceived themselves to have poor social support were more likely to be depressed. They proposed that a similar mechanism for the etiology of depression might exist in schizophrenia as in non-schizophrenic persons.

The most commonly used depression self-rating scales in population studies were developed before the introduction of DSM-III, e.g. the Beck Depression Inventory (BDI) and the Zung Self-rating Depression Scale (Zung-SDS) [11,12]. The Major Depression Inventory (MDI) used in the present study was developed by Bech et al based on the DSM-IV symptoms of Major Depression and ICD-10 moderate to severe depression [13,14]. The MDI includes symptom thresholds as well as duration criteria. The internal and external validity has been reported to be higher than for Zung-SDS [14]. In order to evaluate screening scales there is a need for a "golden standard" and several studies has used Schedules for Clinical Assessment in Neuropsychiatry (SCAN) [15,16]. SCAN incorporates the 10^th ^edition of the Present State Examination and the reliability has been reported to be good [17]. The authors of the scale have reported the sensitivity to be 0.90 and the specificity 0.82 when validating the MDI versus SCAN in a clinical setting [18].

The aim of the present study was to analyze if the psychosocial characteristics associated with depressive symptoms in persons six year after a first episode of psychosis (FEP) differed from the associations found in a population sample.

## Methods

### Population from the Parachute project

The Parachute project is a Swedish multi center study of FEP. It includes all persons from the catchment area who for the first time sought psychiatric help for psychotic symptoms during 1996–1997. Persons who were 18–45 years old and without a dominating substance abuse or diagnosed brain disorder were included in the study. The catchment area covers approximately 20% of the Swedish population. The project integrates an epidemiological approach with intensive psychosocial and medical treatment of a cohort of first episode psychotic patients. It includes a large-scale system of "need adapted treatment" [19], which includes high degree of psychosocial support, lowest optimal antipsychotic medication, participation of families and treatment in normalized and integrated settings. The participating patients were followed for a period of six years with assessments of psychiatric, psychological, social and economic aspects. The project, which is a controlled study, is described in detail in a previous paper [20].

Of the 175 patients included in the project from the start 133 were followed during the complete six-year period. The questionnaire included in this study was sent out to the 133 persons six years after their first psychotic episode and 57.1% (76 persons) participated. Those who participated did not differ in age, gender, country of origin or psychiatric diagnosis from those who did not participate.

### Population from the PART study

The PART study is a longitudinal population study of risk factors and social consequences of mental ill health in the Stockholm County. 19 744 Swedish citizens aged 20–64 years registered as living in the county of Stockholm were randomly selected in 1998–2000. This represents 1.8% of the population in this age group in this area. 10 442 persons (53%) participated in the study. The personal identification number (that all Swedish citizens have) of participants as well as non-participants were linked to the following official registers: income and wealth, sick leave, hospital discharge register (including diagnoses) and disability pension. Participation was related to female gender, higher age, higher income and education, being born in Sweden, and having no psychiatric diagnoses in the hospital discharge register or in the disability pension register. The odds ratios for associations between gender, income, country of origin, education and having a psychiatric diagnosis previously according to the registers, were similar among participants and non-participants (Lundberg et al, manuscript). Participants having had a diagnosis of psychotic disorder in the hospital discharge register were excluded from this study (n = 20).

### Questionnaire

A questionnaire was sent out to the population included in the PART study. The questions included risk and protective factors for mental illness as well as psychiatric symptoms scales [21]. The same questionnaire was replicated in the follow up of the FEP group.

### The following variables were used in the present study

• Demographic characteristics: age, gender and country of origin (Sweden/other).

• Financial problems included the availability to get 14 000 SEK (1 797 USD) within a week, if necessary.

• Working life: The persons were divided in the following two groups: employed/students and unemployed/disability pension/sick leave/early retirement pension.

• Social network: An instrument developed by Unden & Orth-Gomer [22] was used. This instrument is developed from ISSI, the Interview Schedule for Social Interaction [23] Two sub scales were used, AVAT-availability of attachment and AVSI-availability of social integration. The score was calculated according to the authors of the scale.

• Depressive symptoms: The Major depression Inventory was used strictly according to the authors of the scale [13,14]. The scale covers the ICD-10 as well as the DSM-IV symptoms of depression. It contains 12 items, but functionally it has 10 items since two of them contain sub items (restless/subdued and reduced/increased appetite). Each item gives a score from 0–5 based on the following answers: at no time, some of the time, slightly less than half of the time, slightly more than half of the time, most of the time and always. The MDI can be used as a scale for measuring the severity in which the total score is calculated giving a theoretical score from 0–50. A score of 26 is considered pathological according to the authors of the scale.

### Statistical analysis

Simple factorial ANOVA'S were performed using being in the PART population or not as the dependent variable and age, gender and country of origin as covariates. Pearson's correlation was used to see if the demographic and psychosocial variables correlated with depressive symptoms. These analyses were performed separately in the PART and FEP populations. Additionally multiple regression analyses were performed with the scores on the Major Depression Inventory and a cut off score of 26 or more as the dependent variables. All variables were entered simultaneously. Being in the PART population or not was entered as a variable.

## Results

The demographic and psychosocial characteristics of the two populations are presented and compared in table 1. Persons in the FEP population more often had unemployment/disability pension/sick leave/early retirement pension (F = 110.26, df 1, p < 0.001) and financial problems (F = 30.06, p < 0.001). Additionally fewer of them had a sufficient social network ((AVSI; F = 52.26, df 1, p < 0.001) and (AVAT; F = 39.18, df 1, p < 0.001)). They also had higher score on the Major Depression Inventory (F = 25.69, df 1, p < 0.001). The symptoms within the Major Depression Inventory were also analyzed separately. The only symptom that was equally distributed was sleep disturbances; all other symptoms were more common in the FEP population.

**Table 1:** Demographic and psychosocial variables in the FEP population and in the general population sample. The statistical analyses were controlled for age, gender and country of origin (Sweden/other).

	General pop. sample, n = 10 425, %(n)	FEP pop. n = 76, %(n)
Female gender	55.5(5 798)	50.0(38)
Not born in Sweden	10.7(1 120)	15.8(12)
Unemployed^†^	9.7(1 009)	38.2(29)*
Financial difficulties	14.9(1 553)	40.8(31)*
	
	Mean (95%CI)	Mean (95%CI)
	
Age	41.4 (41.2–41.7)	28.5 (26.8–30.3)
AVAT (availability of attachment)	17.7 (17.6–17.7)	16.0 (15.1–16.9)*
AVSI (availability of social integration)	16.2 (16.2–16.3)	12.4 (11.4–13.4)*
Major Depression Inventory	8.8 (8.6–9.0)	15.8 (12.7–18.9)*

^†^Including disability pension/early retirement pension/sick leave, *p < 0.001

When using a MDI cut-off score of 26, 25.8% (17 persons) had a score above the cut-off in the FEP population and 8.0% (783 persons) in the general population sample.

Table 2 presents the correlations between the demographic and psychosocial characteristics and the total score of the MDI. In the general population sample all demographic and psychosocial variables were found to be associated with the total score of the MDI. In the FEP population not born in Sweden and being younger were not associated. The gender associations were different in the two samples, while female gender was associated in the general population sample male gender was associated in the FEP population.

**Table 2:** Correlations between the demographic, psychosocial characteristics and total score of the Major Depression Inventory in the FEP population and in the general population sample.

	General pop. sample N = 10 425	FEP pop. N = 76
Not born in Sweden	0.11***	0.12
Age	-0.13***	-0.01
Female gender	0.13***	-0.21*
Unemployment	0.14***	0.23*
Financial problems	0.27***	0.35**
AVAT (availability of attachment)	-0.32***	-0.48***
AVSI (availability of social integration)	-0.31***	-0.41***

*p < 0.1, **p < 0.01,***p < 0.001

Separate multiple regression analyses were performed in the two samples with the Major Depression Inventory total score as the dependent variable. In the general population sample the adjusted R square was 0.21 (SE 8.7) and in the FEP population 0.28 (SE 11.4). In addition an analysis was made were general population/FEP population was inserted as a variable. The result is presented in table 3, and shows that the correlation between being a person having had a FEP and higher scores on the Major Depression Inventory no longer was present when the other variables were taken into account. Adjusted R square for this analysis was 0.21 (SE 8.7). A similar regression was performed entering depressed/not depressed using a cut-off score of 26 and the result is also presented in table 3. Adjusted R square for this analysis was 0.12 (SE 25.5)

**Table 3:** Multiple regression analyses with total score on the Major Depression Inventory and a MDI cut off at 26 as the dependent variables.

	MDI Score Beta _Stand._	MDI Cut-off Beta _Stand._
FEP pop. vs. General pop. sample	0.00	0.00
Not born in Sweden	0.04*	0.04*
Age	-0.16*	-0.07*
Female gender	0.13*	0.08*
Unemployed	0.10*	0.09*
Financial problems	0.10*	0.09*
AVAT (availability of attachment)	-0.15*	-0.19*
AVSI (availability of social integration)	-0.24*	-0.09*

*p < 0.001

## Discussion

The main finding of this study was that the association between having suffered a FEP and self-reported depressive symptoms/depression six years later disappeared when a negative social situation was taken into account.

Not surprisingly, persons with a previous FEP had a higher representation of unemployment, financial problems and insufficient social network, which have been reported in other studies [24-26]. In the general population there was an age and gender effect, females and younger age had an overrepresentation of depressive symptoms. This was not seen in the FEP follow up group where age had no effect and being a male was slightly over represented. This is in agreement with a study by Zisook et al [9]. Not born in Sweden was associated with depressive symptoms in the general population sample, but not in the FEP population. This could have been due to low numbers in the FEP population.

The non-participation rate was high in the general population and persons with severe psychiatric disorders most likely did not respond to the enquiry. However, the associations between gender, income, country of origin, education and having a previous psychiatric diagnosis was similar among participants and non-participants. The FEP group also had a high non-participation rate, although the distribution of age, gender, country of origin and psychiatric diagnoses were similar among participants and non-participants. The general population sample was an urban population while the FEP population was from areas all over Sweden, which might have affected the result.

The strengths of the study were that the FEP group was a total population, followed over six years and having received treatment according to a "need-adapted approach". Moreover, the instrument in use, the MDI has been reported to have a higher internal validity than Ham-D_17 _and Zung-SDS [13,14]. The sensitivity and specificity have been above 0.80 when the MDI was compared to clinical interviews using SCAN [18].

The results of this study fully agree with the goals of WHO [27]: Links need to be established between mental health services and various community agencies at the local level so that appropriate housing, income support, disability benefits, employment and other social service supports are mobilized on behalf of patients and in order that prevention and rehabilitation strategies can be more effectively implemented. Following these recommendations would most likely decrease the rates of depressive symptoms in former FEP person with a secondary positive effect on their quality of life in general.

## Conclusions

Having had a first episode psychosis six years earlier had no association with depressive symptoms/depression when a negative social situation was taken into account.

## Competing interests

The authors declare that they have no competing interests.

## Authors' contributions

YF, SL and JC participated in the data collection. YF performed the statistical analysis. All three authors wrote and approved the final manuscript.

## Pre-publication history

The pre-publication history for this paper can be accessed here:


